# “Are They Going to Play Nicely?” Parents’ Evaluations of Young Children’s Play Dates

**DOI:** 10.1007/s10826-022-02499-4

**Published:** 2022-12-13

**Authors:** Alison J. Lacey, Robin A. Banerjee, Kathryn J. Lester

**Affiliations:** School of Psychology, University of Sussex, Brighton, UK

**Keywords:** Play date, Children, Qualitative, Supervision

## Abstract

Over the last 20 years, opportunities for young children to engage in self-directed free play with peers outdoors or during the school day has dramatically declined. Consequently, it is likely that play dates, defined as pre-arranged social contact at home, have become increasingly significant play environments for children. Preliminary research suggests that play dates are positively associated with young children’s social and emotional development, but that access can be strongly influenced by parental social networking priorities. However, little is currently known about the nature and frequency of play dates, the types of play children engage in, or the impact of parental management and supervision on children’s play in this context. Exploratory qualitative research is essential to understand the extent to which parental gatekeeping may limit opportunities for children and families perceived to have low social capital, and to begin to define the nature and content of young children’s play dates more broadly. Parents of children aged 5-6 years old took part in a semi-structured interview to describe common practices and attitudes (*N* = 11). Inductive thematic analysis indicated that play dates are complex play environments that parents associate with a range of social, emotional, and cognitive benefits for children. However, close parental monitoring and supervision may limit the extent to which play dates provide opportunities for self-directed free play. Findings also show that access to play dates is strongly influenced by parents’ motivations to enhance children’s social status which restricts access to some children. Implications for future research are discussed.

## Highlights

- Play dates are important contexts for children to experience relatively unstructured free play.
- Play dates may provide unique opportunities for independent imaginative play.
- Access is not universal with some children at risk of exclusion.
- Play dates are often effortful and stressful for parents.
- Parental supervisory styles alter the quality and nature of children’s play.

Play dates are early social events that lie at the interface of family and peer relationships. They mark the beginning of the child’s entry into the social world and have been associated with social and emotional skill development in preliminary studies (Ladd & Hart, 1992). Play dates are common, normative experiences for young children leading up to and during primary school (4–11 years of age in the UK). The term ‘play date’ typically refers to play between peers in the family home: an environment that combines a young child’s dominant family system with the emerging influence of friends, although play dates may also occur in other locations, for example parks or playgrounds. Play dates are rich environments in which to explore the connected yet distinct systems of the young child’s social world (Hartup, 1979) and to observe how children and parents negotiate increasingly complex social environments.

Over the last 20 years there has been a steady decline in allocated play times during the school day for children in the UK (Baines & Blatchford, 2019) and for informal play outdoors (Bendelow and Mayall, 2002). Consequently, it is likely that play dates are increasingly important environments to observe social play in young children. The practice of social peer play, analogous to play dates, can be found in both historical and anthropological records (Lancy, 2014). Play dates are ubiquitous: during the autumn term in which this study was conducted (September–December 2018) play dates were mentioned in over 500 discussion threads on the UK parenting forum, Mumsnet, including active threads entitled: ‘Play dates—what is the protocol?’; ‘Should I ask for a play date?’ and ‘Anxiety and play-dates’. However, despite this ubiquity, relatively little psychological research has explored the quality and nature of children’s play during play dates, or the extent to which these early play experiences may influence children’s friendships and social networks.

Research findings to date suggest that play dates may be an important environment for children to practise social relationships and to experience relatively autonomous free play from the secure base of the family home (McAuley & Rose, 2014). For example, frequency of play dates has been associated with increased peer acceptance and decreased peer rejection for children aged between 4-6 years of age in cross-sectional research (Ladd & Golter, 1988; Ladd & Hart, 1992). Play dates may be particularly useful environments for children with additional needs (Chambers & Horn, 2010; Frankel et al., 2011), although we know these experiences tend to be less common for children with social or behavioral problems compared with their peers (Frankel & Mintz, 2011). A sociological study exploring parent and teacher attitudes to play dates in New York suggests that unequal access to play dates may be partly explained in terms of social capital and parent networking priorities (Mose, 2016). Mose’s analysis of 41 interviews with parents and teachers demonstrates that play dates are important markers of social advantage and are often the vehicle through which parents seek to enhance and preserve social status through intentional networking and tightly regulated and controlled play partners (Mose, 2016).

These findings indicate that play dates are complex social events for parents and children, with significant cultural and social associations that may influence the quality and range of children’s play as well as access to these early play experiences. The relative lack of research in this area is, therefore, surprising, especially when we consider that they are an important and common context for children to play with their peers. In a study of 421 children aged between 5-12 years old, 59% of children rated play dates at home or at a friend’s house as their preferred play environment making home-based social play the most popular play category overall (Tandy, 1999). In another study involving analysis of photographs taken by children of their most common play environments, 53% of photographs were inside the home (Veitch et al., 2006).

There are well-established associations between young children’s access to peer play and social, emotional, and cognitive skill development (Gleave, 2009), although these associations have not yet been widely studied within the play date context. Child-led free play, in particular, has been associated with the development of resilience, mental well-being, and openness to learning and creativity (Lester & Russell, 2008) and social interactions with peers have been described as the most important context for learning and the development of emotion regulation (Galyer and Evans, 2001). Peer play is also an important predictor of sociometric status with increased socially active leisure time spent with friends positively correlated with high peer status (Östberg, 2003).

Play is a priority within early years’ education strategies (Beyer et al., 2015) and play dates are likely to be an important context for children to develop the skills and social and emotional foundations that underpin successful transition and adjustment to school. Friendships developed through play protect against anxiety, stress, and loneliness (Booth-Laforce et al., 2006) and support children’s transition and adjustment to school by creating strong social networks and via the acquisition of social skills and values (Bukowski et al., 1996). The ability to make and maintain positive peer relationships is a key indicator of school readiness. Research shows that observed play competencies at home positively predict prosocial behaviors, classroom motivation, task persistence, and autonomy (Fantuzzo & McWayne, 2002).

We do not yet understand how parental management of play dates may influence the quality of children’s play. However, previous research suggests that variability in parental supervision during play is likely to moderate the social, emotional or cognitive benefits for children. For example, high levels of parental supervision are associated with increased structured and formal activities and reduced unstructured play (Bryant, 1985). This is important when we consider that unstructured play activities predict improved social-emotional functioning (Bryant, 1985; Ladd et al., 1992) and enhanced perspective-sharing and peer cooperation (Rogers, 2012). Highly supervised play has also been associated with a diminished ability for children to recognize potential play affordances (Ergler, Kearns, and Witten, 2013) and reduced free and imaginative play (Ladd et al., 1992). Children’s social relationships are thought to be qualitatively altered by the presence of parents (Lollis et al., 1992) partly due to a lack of opportunity to independently manage challenging social interactions (Bryant & DeMorris, 1992). Children themselves value play opportunities away from the gaze of adults: a qualitative study of 123 7-10-year-olds found that autonomous play with peers helped children view themselves as simultaneously capable and care-free (Rogers, 2012). However, child-led free play may not be appropriate for all children. Research exploring the play behaviors of children with additional needs or low social competence identifies direct parental management of play dates as optimal, suggesting that child-led free play may require a certain level of baseline social skills and competence. These studies include advice for parents to select socially competent play partners for play dates (Chambers & Horn, 2010), and to actively manage the play environment (Harris, 2015; Jull & Mirenda, 2011).

The extent to which adult involvement supports or interferes with play for typically developing children, however, has not yet been fully explored and is especially relevant in the context of play dates which generally take place inside the home with at least one parent present. The Vigilant Care Model of parental supervision provides a useful theoretical framework to understand the role of parents during children’s peer interactions (Omer et al., 2016). The model describes three levels of appropriate parental involvement depending on context: open attention; focused attention; and active protection. Open attention is the default approach in most circumstances and is thought to foster an open, non-intrusive, authoritative environment. At this level parents are available and responsive to the child without being unduly involved in their activities. Focused attention and active protection approaches describe graded responses to distress or difficulty. Focused attention might involve parents offering verbal support or guidance, for example, whereas active protection is likely to necessitate an intervention to directly manage the interaction or situation. The Vigilant Care Model encourages parents to sensitively scaffold their child’s environment to allow autonomy and agency. Parents who adopt open attention supervision styles during play dates may be more likely to increase opportunities for children to engage in self-directed play that we know supports healthy social and emotional development (Tremblay et al., 2015). Variability in parental supervision and monitoring during play dates, therefore, is likely to be an important, and previously neglected, aspect of young children’s peer relationship development.

Exploratory qualitative research in this area provides important insights into cultural understandings associated with play dates from the perspective of parents as well as providing a framework for the ‘mapping’ of the play date experience to support hypotheses for larger scale quantitative work. This study is the first of its kind to qualitatively explore the nature of young children’s play dates from the perspective of parents of children aged 5–6 years old in their second year at school in the UK. One of our primary areas of interest was the extent to which play dates are associated with friendships and social capital during children’s transition to school. Having completed a year at school we felt that parents of children in Year 1 (children aged 5–6 years old) would be able to reflect on their child’s experiences of play dates both before and since starting school and to consider the impact of these experiences on their child’s social development over this period. This study explores variability in terms of the quality of children’s social play during play dates focused around four key research questions: What are play dates and what families have access to them? How do parents feel about play dates? What do children do during play dates? How do play dates change in association with children’s development?

## Method

### Participants

Participants (*N* = *11)* were recruited via advertisements in local on-line parenting forums serving a major town in the south-east of England during December 2018. A provisional sample size of 10–12 was determined prior to data collection based on samples used in previous research (Vasileiou et al., 2018). This initial estimate was then reviewed following preliminary analysis. Given the high level of consistency between participants’ accounts of play dates, we agreed that the data were sufficient to determine theoretical insights based on principles of theoretical sufficiency rather than thematic saturation (Braun & Clarke, 2019). This judgment of theoretical sufficiency was based on interpretative, situated, and pragmatic considerations, recognizing that the concept of data saturation is not always consistent with the values of thematic analysis (Braun & Clarke, 2019).

Of the 11 parents who took part, ten (91%) were mothers, and there was one father. Most participants (91%) were either married or co-habiting, and one was single. Participants represented a range of socio-economic backgrounds with seven participants (63%) reporting household income above the national median (£28,400) for the UK in 2018 (Office for National Statistics, 2019) and five (45%) having obtained an undergraduate degree or higher. Four (36%) participants described themselves as ‘stay at home’ parents, four worked part-time, two were self-employed and one participant was employed full-time. All participants described themselves as White British in an area with an above national average White British population (85% compared with 79%) (Office for National Statistics, 2012).

Parents were asked to reflect primarily on the experiences of their Year 1 child (focus child). Of the 11 focus children eight (72%) were White British and three (27%) were described as having mixed/multiple ethnic backgrounds. This represents a slightly greater ethnic diversity than the local and national population. Two children (18%) had no siblings, four children (36%) had one sibling, three children (27%) had two siblings, and two children (18%) lived in households with three or more siblings. Five (45%) children were female. The age of focus child ranged from five years four months to six years one month (*M* = 5 years 9 months). All children were assessed by their parents to be typically developing, although one child had a diagnosed speech delay.

### Procedure

One-hour telephone interviews were conducted, and audio recorded, by the first author using a semi-structured interview guide. To avoid introducing prior assumptions about play practices and norms, the term ‘play date’ was only used if parents introduced the term themselves. During the interview, parents were asked to reflect on their child’s social play at home with children from outside their immediate family before and since starting school and to consider developmental changes over time. Questions were designed to address the four research questions identified as important gaps in current play date literature. The aim of the study was to generate data rather than test hypotheses. Consequently, open questions were used to encourage participants to describe their experiences in as much detail as possible.

All interviews were digitally recorded and transcribed verbatim. All identifying information was removed during transcription and each participant was given an alias. Aliases are used to identify individuals throughout this report, followed by M or F to indicate whether the focus child was male or female.

### Data Analysis

Transcribed interviews were analyzed following the six stages of thematic analysis as described in Braun and Clarke (2006) using Nvivo12 Pro. Thematic analysis is a flexible method accommodating a range of theoretical approaches and in this study we adopted a realist, inductive approach to data analysis (Wiltshire & Ronkainen, 2021). Inductive thematic analysis is an active process: themes do not emerge from the data, rather they are actively selected, edited, and interpreted by the research team. As such, it is important to acknowledge the active role of the researcher in the analytic process. All three authors are parents with direct experience and understanding of play dates. This personal experience supported the building of rapport with participants and facilitated the sharing of experiences from a position of mutual understanding. In addition to these benefits, we were mindful that our own experiences could influence our interpretation of the data. To address these concerns, we took active steps to minimize this risk, including holding fortnightly meetings throughout data collection and analysis to discuss our own assumptions and experiences, and to challenge interpretations of the data in the context of these experiences.

Inductive coding is an iterative process of reading, checking, and applying consistent codes (Percy et al., 2015). To ensure that the perspectives and experiences of each participant were accorded equal priority, non-hierarchical initial codes were generated for each transcript. Codes were then synthesized into themes across the dataset in a recursive process that included frequent checking back to the original data. Once initial themes had been identified, emerging patterns were checked for consistency or divergence of experience.

Finally, identified patterns were organized into five level two super-ordinate themes which represent the latent themes and interpretative findings of the first author (Braun & Clarke, 2006). To ensure credibility of findings, all three authors met fortnightly to consider coding and thematic structure and to reach agreement that the identified themes provide an accurate, coherent and distinctive perspective on the dataset.

## Results

Table 1 sets out the main themes and sub-themes identified during thematic analysis, along with a brief description.

**Table 1:** Summary of themes

Theme	Description
1: Play date norms	Describes common play dates norms, including reflections on the homogenization of children’s play. Also describes common practical barriers to access.
Prototypical play dates	
A proper ‘do’	
Practical barriers	
2: The play date community	Explores the community of children and parents likely to be involved the play date network, including families perceived to be ‘like me’. Also explores the social networking purpose of play dates to enhance and expand children’s social status.
Families like me	
The nice kids	
Extending networks	
Classmates	
3: Parental Ambivalence	Explores parental ambivalence about play dates, including parents’ perceptions of the value of play dates in supporting children’s social and emotional development and outlines perceived costs for parents.
Social and emotional benefits for children	
Benefits for parents	
Parental stress and anxiety	
4: Types of Play	Describes the range of play typical during play dates, in particular the extent to which play tends to be child or parent led. Also considers significance of screens and technology
A unique play environment	
Unstructured free play	
Parent-directed play	
Screens and technology	
5: Play date management	Explores variability in play and parental involvement in play dates during the transition from pre-school to primary school.
Frequency and initiation	
Parental supervision	
Play styles	

### Theme One: Play Date Norms

#### Prototypical play dates

There was consensus among parents about the typical structure and content of play dates which were described as pre-arranged, loosely supervised after-school play between two primary aged children at one of the children’s homes and which generally involves an evening meal. All parents in the sample reported hosting play dates at home.

I’d say it’s normally more after school: pick up, come to the house, have a play, have something to eat, and get picked up later, that sort of thing. The first part is more the play element with the imaginative play, maybe the Lego, maybe a bit of football in the garden and then it’ll be food and a more chilled out time because they’ve had that excitement. (Isabelle, M)

Play dates also frequently included a trip the park, especially during the summer: “If the weather was OK, I’d think, ‘right I’m going to kill an hour of this play date in the park’… let them, you know, have a good old run around” (Katy, F). Parents commented that the widespread use of the term ‘play date’ had contributed to the homogenization of play at home with parents feeling that children experienced less variety than was the case for previous generations: “Generally speaking, I think that, yeah, play dates are often carbon copies of one another.” (Katy, F)

#### A proper ‘do’

The word ‘play date’ was identified as a universal, if rather unpopular, term: “I don’t actually like that word, but it is what I use” (Rebecca, M). All parents used the term regularly to describe children’s play at home: “Everyone uses it” (Michelle, F) with a number of parents noting that the term itself had contributed to what they perceived as a more formalized experience of social play at home over the past 20 years or so, making it a proper ‘do’ (event) rather than a spontaneous activity. Participants felt that the term conferred a sense of exclusivity, with connotations of an official date between previously determined partners: “It [the word play date] makes it a proper ‘do’. It’s a proper thing instead of a more relaxed, ‘can I have a friend round mum?” (Vanessa, M). Another parent commented, “it sounds a bit silly, a bit over the top. A play-*date* Like an official date.” (Natalie, M).

In line with the perception of play dates as semi-formal social engagements, parents also reported a strong reciprocity norm, with most families feeling indebted until the favor had been returned: “There is—it’s unspoken, it’s an unspoken expectation that you should have that friend in return back to your house. I’m not sure if it’s just me that feels that but it’s definitely there.” (May, F). “You kind of feel anxious until you’ve actually done it, you know” (Sarah, M).

#### Practical barriers

Most parents reported that opportunities for play dates are often restricted by work and children’s after-school activities. Many parents reported feeling that their children’s extra-curricular commitments were already too much and that they struggled to fit play dates in, “I mean my children do too much. They do an awful lot of extra-curricular activities—it’s a long day and then after school they might have… drama and ninjas and all sorts of silly things” (Vanessa, M). “There’s so much in and out of school that happens now. I mean it’s almost a bit bonkers really that they do so much” (Michelle, F).

### Theme Two: The Play Date Community

#### Families like me

Play dates occur most frequently between families with existing social relationships or who are perceived to be “like me” (Tina, F) and participants were more likely to report having play dates with families perceived to share similar values. Although only two parents explicitly mentioned social class as a barrier, this was implicit in other interviews where a preference for play dates between “like-minded” families was reported. “I would only really allow him to go to play date houses that I know the parents, know the children, and feel comfortable and confident with them.” (Natalie, M). “Snobby is not a nice word, is it? But… as long as I thought the parents that they were going to were nice and—like-minded is probably the best word,” (Vanessa, M).

#### The nice kids

Although both parents and children contributed to initiating play dates, parents tended to actively encourage play dates with children perceived to be a good influence. The most likely children to receive invitations were those with a reputation for behaving well and with whom parents wanted to encourage a more lasting friendship. “As long as they have manners. That’s a really, really big thing” (Tina, F).

They’re just nice kids, you know. You can tell who is a nice child. You hear from what they say in school, you know, who they’ve played with and…who has behaved in class. You kind of pick up quite quickly who the good children are and who the naughty ones are—you see them with the parents and—you sort of work out who are the good ones. (Michelle, F)

Parents identified child characteristics that might prevent them from extending play date invitations. These included children with a reputation for being naughty or with challenging behaviors; those with special educational needs for whom they believed play dates might be more challenging; and those from unknown or lower SES families.

It only probably takes me once to have a kid from hell round [laughing]. And I’m like, ‘right that one’s not coming over again’. I’m going to have to invent excuses for that person not coming around to play. (Katy, F)

#### Extending networks

Parents reported using play dates to actively manage their child’s social circle by extending their child’s social network and helping them integrate more effectively within the class. “[I might invite different children] to extend his comfort zone and build the relationships because I think that’s how you can grow.” (Natalie, M)

Parents also described play dates as useful opportunities for children to “work out where they fit” (Katy, F) and as an early intervention to smooth over any emerging friendship difficulties. “There were one or two children that I arranged more play dates with…to try and sort out the problems she was having in the class” (Michelle, F).

#### Classmates

Most parents reported strong social group identification between members of the same class. Children in other classes within a year group, and friends from outside of school, were less likely to be considered part of a child’s network and consequently less likely to be considered for play dates.

We were outside in the playground after an after-school club, and she (daughter) was on the monkey bars and the little girl behind her was somebody that she’d been very friendly with at nursery. I said, ‘why didn’t you talk to her?’ And she said, ‘she’s not in my class, I don’t know her anymore’. (Katy, F)

### Theme Three: Parental Ambivalence

All parents considered play dates to be valuable social opportunities for their child that help support the development of social and emotional skills, including sharing, negotiation, resilience, and compromise. However, they also reported high levels of stress for themselves including frequent social comparison anxieties and feelings of effortful social presentation.

#### Social and emotional benefits for children

Parents associated play dates with a range of benefits for children including increased confidence, learning to play more independently, sharing, practising manners, negotiating positive friendships, resilience, excitement and anticipation. “They definitely strengthen the relationship which is obviously why we go through—I mean you become more confident playing and you can see relationships growing” (Natalie, M).

It’s really important for their social development. I think everything is learned through your interaction with your peers and with your friends. So, learning to share, learning your manners, learning how to—how to create a game. You know, how to decide who is going to be who… so quite often the role-play things that are kind of left to the side in the playroom, they come alive when they have friends over and they’re playing shops and they’re dressing up. (Michelle, F)

Parents reported that play dates support children’s peer relationships by strengthening existing friendships, providing opportunities to establish a stable peer network, and learning conflict resolution strategies: “I think they become closer in school. I know that in the past they have played with each other for the whole day [at school] because of the play date.” (May, F). “From reception [first year at school in the UK] I was very conscious to have play dates. I think it is really important. I think it gives them a bit of confidence and just broadens their friendships really” (Caitlin, F).

Sibling relationships were identified as an important factor influencing the extent to which play dates were perceived to benefit children. Parents tended to consider children with older siblings to be more equipped to deal with the social demands of play dates whereas children without siblings were thought to benefit more from the freedom and novelty of play dates. “I think because he has a big sister, he’s been able to say, ‘come and see my room’, and he would show them round because he would see what his sister did when her friends came” (Vanessa, M).

You definitely notice that the children who have siblings, even if they’re the elder or the younger sibling—both ways— because they have this sibling rivalry, they are more adept at school emotionally, I think. They’re more knowing how to get their needs met because they’ve had to fight for that and, you know, a single child hasn’t had that exposure therefore the play is different, and their behavior is different. (Natalie, M)

#### Benefits for parents

Benefits to parents of hosting and allowing their child to attend play dates included the opportunity to monitor their child’s social interactions, benchmarking their child’s cognitive and social development against their guest, enjoying some free time while their child was being entertained; and the enjoyment of seeing their child playing in a safe environment. “You see how other kids are and you think, you know…‘well mine’s not so bad’, you know” (Sarah, M).

It gives you more of an insight into how your child interacts with other children. Yeah, so I definitely think it is important. And then if there’s something that they struggle with you can perhaps help to—help them to feel more comfortable or overcome anything. (Caitlin, F)

Parents also reported that play dates are an important opportunity for adults to develop social networks and can be a valuable informal childcare resource. Parents commented that part of the motivation to arrange play dates was for parents to socialize together “so we both [parent and child] get something out of the situation really” (Tina, F). Consequently, play dates were valued as opportunities to strengthen social networks and close friendships with other parents. “I think you get a lot closer, especially if your children get on and play nicely together, you sort of want to…spend more time with them” (Michelle, F).

I think it’s brought us closer which I think is important to have that because, you know, I know at times they’ve relied on me to help them out and I’ve relied on them to help me out. And I think it’s good for the children to observe your relationships with the parents too. (Isabelle, M)

Most parents reported valuing the opportunity to build relationships with their children’s playmates and to be part of that child’s wider social network. “You have a bit more of a bond with other people’s children once they’ve been to your house” (Caitlin, F).

#### Parental stress and anxiety

However, parents reported that play dates often feel like hard work and require significant effort both in terms of the extra supervisory responsibility during the play date itself and organization and preparation in advance. Play dates with new guests were more likely to cause parents’ stress: “Are they going to play nicely?” [laughing] (Michelle, F). “I’m always quietly nervous before they come because you feel like it’s more of a commitment and it’s going to take more of your time up whilst obviously trying to juggle making dinner and the other children.” (May, F).

Parents also reported feeling concerned about what the guest child and their parents would think about their home with one participant reporting that she knew another parent who felt unable to host play dates due to social comparison worries. Tidying in advance of a playdate and concerns about what the guest child would think of the environment were also drawbacks for most parents: “You know, is the other adult judging you? Like, ‘oh, you do it that way’ or ‘oh, you’ve got your house like this’” (Sarah).

I think you spend most of your time as a parent—even though you know not to, looking around comparing, thinking, ‘is this OK, is it OK?’… I’ve got a friend who doesn’t have any play dates and she feels incredibly guilty—she deems her house too small. (Vanessa, M)

Parents also reported feeling anxious about making sure the play date was sufficiently fun for the guest: “I don’t want someone to come to our house and say, ‘that was really boring’… So, it’s this kind of balancing the whole thing and hoping that it has been exciting” (Isabelle, M).

Not being invited for play dates and feeling left out of the class social network was a concern for some parents who reported that play dates could be alienating. Other parents described having play dates as an expectation, which often felt like an additional pressure in the context of busy family life. The social networking element of play dates contributed to this pressure to have play dates, with parents feeling concerned that children might be left behind socially if their child was not included in the play date network: “If my child wasn’t invited—well, why? You know, there was certainly that playground chat of, ‘well why hasn’t mine been invited around to that one’s house?’” (Sarah, M). “There is a massive pressure generally which is bizarre” (Vanessa, M).

Most parents reported conflict between children as a challenge they preferred to avoid. Parents tended to intervene with verbal guidance in minor disputes if the conflict did not quickly resolve and most reported feeling reluctant to directly address poor behavior from the guest: “I don’t like telling children that aren’t mine that it shouldn’t be a certain way. I try to avoid telling them off.” (May, F). “I tend to cut the guest a lot more slack probably than I should because they’re the guest” (Rebecca, M).

### Theme Four: Types of Play

For most children, play dates are times of relatively unstructured, minimally supervised play. Imaginative role-play games are common with parents reporting that play dates offer unique opportunities for creative dyadic play not typically observed in more structured environments.

#### A unique play environment

Parents reported that children play very differently during play dates than in other contexts with the opportunity for close dyadic play offering a new perspective on toys and games: “It’s all quite positive because they play quite a lot, they do things that they wouldn’t necessarily do on their own… he wouldn’t ever dress up so obviously his friend would dress up and then he would” (Tina, M).

They play different games, and they do things in different ways than when they play on their own. It’s nice to see how—toys that they’ll never play with on their own on a play date will suddenly come to life. (Vanessa, M)

Parents considered play dates to be unique social experiences that foster the development of skills not necessarily available in other environments. In particular, learning the roles of guest and host, focusing and extending school playground play, and engaging in imaginative role play parents felt would not have occurred in other contexts. Parents also valued the opportunity for children to strengthen friendships with specific friends in a more intimate environment than at school.

I think it’s really important. I think in school it can be—there’s a lot of people, you know, they’ve got 30 kids in the class and they’re all wanting to play their own games so there’s quite a lot of jostling for attention and who’s going to do what. When it’s just one on one, or there’s like three of them, they can have—they can play more—they can play games more suited to what they want to do. (Isabelle, M)

Parents of only children described being motivated to arrange play dates to provide a unique and important socialization experience. These parents described play dates as opportunities to experience the dynamics of peer conflict, rivalry, and sharing that is not possible to replicate fully at school.

I think it’s important because other people have siblings, and they see that interaction but because he’s an only child—so obviously you see a lot of siblings hitting each other [laughing]… So, I think it’s important to see that variation of how people interact and how they are. (Natalie, M)

#### Unstructured free play

All parents reported that play dates are mainly times for unstructured, imaginative free play. Typically, children are encouraged to leave the immediate supervision of the parent and manage their own play.

It’s actually quite nice for them to go off grid a little bit and not have a grown up saying, ‘we’re doing this next and we’re doing that’. I think that’s the beauty of a play date. I kind of think that’s part of what they learn—they learn what they do and don’t want to do. (Katy, F)

Frequent opportunities for free play during play dates was described as important to enable children to learn the skills necessary to sustain high quality play: “It’s amazing. Playing is not an easy thing. It’s something they have had to get used to doing over time and when they’re around other children playing, it really does teach them how to” (May, F).

#### Parent directed play

Parents reported structuring play activities for younger children or with children perceived to be more challenging or in need of more supervision. Although parents agreed that children generally preferred unstructured activities, they were more likely to structure play if they felt the other parent would expect it: “I’d been to their house, and they had—their mum had prepared lots of activities. So, I thought, ‘oh, OK, I guess this is what is expected’ kind of thing. Or what this child does” (Rebecca, M).

So, if I think that it’s a play date that I would need to manage (if that’s the right word?) a bit more I would—we’d make cakes or if I thought that a play date wasn’t going very well, or that they weren’t very happy or seemed to be bickering or something, I would definitely steer over to, ‘let’s bake, or let’s paint’. (Vanessa, M)

#### Screens and Technology

In general, parents tended to avoid the use of technology during play dates, although most did allow children to play computer games or watch TV if they felt they needed ‘downtime’. As children got older parents felt more pressure to allow access to screens which they felt ultimately limited opportunities for creative, imaginative play. [Speaking about an older sibling, aged 8] “I think it was very much play based really, toy based, and now as they’re getting older it’s more technology” (Sarah, M).

### Theme Five: Play Date Management

#### Frequency and initiation

Most participants reported that both parent and child-initiated play dates increased sharply during the first year at school as parents actively managed invitations to try and establish their child positively within the peer group and children became more active social agents. Although play dates during pre-school tended to be based primarily around the parent’s existing friends, parents reported that starting school marked the beginning of the child’s own efforts at social initiation and integration. Parents tended to remain involved in their child’s social relationships and school transition by actively encouraging and initiating play dates throughout primary school. The reception year (first year at school in the UK for 4–5-year-old children) was highlighted as a particularly important time for parents to support peer integration with most children having play dates at least once a fortnight during this time, although frequency tended to be lower for families with work and extra-curricular commitments, or where siblings were close in age. “It was me who often initiated. Trying to work out who she was playing with in school and then trying to get the friend to come over so she could kind of make friends.” (Caitlin, F). “A lot of the reception year was exploring different options, I suppose, and now this year it’s very much the same set of people that come over.” (May, F). Play dates immediately before starting school were described as an important way for parents to support successful school transition so that “rather than just throwing them in they kind of already felt safe and they already knew someone” (Tina, F).

#### Parental supervision

High levels of direct parental supervision during play dates tended to decrease following the first year at school as children became more autonomous within the home. Most parents reported indirectly supervising children using open attention, facilitative parenting strategies by allowing children to play out of sight and reach to allow them freedom to manage their own social event. Indirect or open attention styles of supervision were felt to be commensurate with school age and most parents changed from direct to indirect styles (i.e., from highly supervised to more loosely monitored play environments) during the reception year [age 4-5 years old, pre-Kindergarten in USA]. “He’s much happier to go off on his own—well with his friend without me.” (Rebecca, M) “Once we get into the house then I kind of just leave them to it within reason” (Katy, F).

Parents reported using more direct supervisory styles during structured activities or if the guest child was new, or had challenging behavior: “If there was a child that I knew had certain behavioral traits I would be more—dropping in more.” (Caitlin, F). “I would be more of a helicopter parent in that play date” (Natalie, M).

However, very few parents left the play entirely down to the children with most reporting guiding children’s play by suggesting activities and/or leaving out toys or equipment they felt might inspire positive play: “I’ll kind of suggest things. We’ve got various toys and games that are good for playing with other children.” (Rebecca, M). “We’ll already maybe have the train set out and, you know, I’ll say, ‘do you want to play together with this?’” (Vanessa, M).

After the first year at school, parents reported that it is unusual for the non-host parent to stay. During pre-school, parents of both children tended to be present, but this changes during the first two terms at school unless both parents have younger children.

It’s gone the totally opposite way now. My friend said the other day—she said, ‘and she stayed!’ She invited—I don’t know what it was, she invited someone to come and play—well, the child or so she thought and then she was quite confused when the mum stayed…That’s not the done thing! (Vanessa, M)

#### Play styles

Most parents reported that during the second year of school (Year 1 in the UK) children’s ability for sustained focused play improved markedly, and that they demonstrated improved social interactions and conflict resolution. “They can be absorbed in a game for longer. Their attention span has massively improved, so they are lost for longer in a game.” (Vanessa, M). As children develop, parents predicted that they would become less interested in imaginary play and more focused on screens and technology with the age range 5-8 years highlighted as optimal times for free, imaginative play with peers at home.

I will be quite sad when they stop doing all the role-playing and all the imagination games… It’s just starting to happen with [older sibling] who is 8. You can just see—I think another year and that’s going to have finished and I think that will be quite sad because I think that’s such a lovely part of childhood. All those imagination games. (Michelle, F)

## Discussion

This study qualitatively explores young children’s play dates—an important, common, and under-researched play context—from the perspective of parents. Results indicate that play dates are regarded by parents as significant social events that positively influence children’s social and emotional development during the transition to school. Parents associated play dates with enhanced social status and social capital, describing them as significant opportunities for children to practise important social skills and to establish themselves securely within the wider social group at school. Importantly, parents felt that play dates offer unique play experiences unavailable in other contexts. For example, learning to share possessions, manage conflict, practise the role of guest and host, and participate in extended imaginative play. One parent described play dates as an essential part of learning the art of play, acknowledging that play is not always instinctive and is a skill children need to develop. Parents of only children were especially likely to describe play dates as important, describing them as opportunities to observe and participate in the challenges of sibling and peer relationships in an informal context.

Parental management of children’s play was associated with variability in the quality of children’s play. Although most parents recognized the value of imaginative, child-led play, most reported keeping track of play and intervening when children became bored or fractious, and suggesting or guiding play to make sure guests were kept entertained. Parents were also more likely to allow child-led play with children they felt were already adept at this kind of activity. These findings align with previous research that suggests that child-led free play requires a certain level of social competence and may not be appropriate for children with less well-developed social skills (Harris, 2015). However, parents’ tendency to actively manage play was not solely determined by perceived child play competence. Although parents tended to feel that children enjoyed just “going off” and playing, they reported an expectation to provide a valuable and fun experience for their guest which made them likely to closely monitor and keep track of children’s play. Again, these findings align with previous research that suggests that play dates are not neutral play events but are often invested by parents with an imperative for effortful cultivation of children’s social lives and, as such, are part of the active work of parenting (Mose, 2016). Theoretical models of effective parental scaffolding show that transfer of responsibility from parents to children is a crucial part of children’s social development, especially during significant developmental shifts i.e., starting school (Neitzel & Stright, 2003). In play dates, this transfer of responsibility may conflict with social and cultural expectations for parents to provide a harmonious and valuable learning environment for children. In particular, these expectations may encourage over-management of children’s play which may prevent children from managing conflict or boredom, or planning and developing games for and by themselves (Mose, 2016).

We know, for example, that conflict is common and normal during peer play: one study reported an average of eight separate conflicts per hour during play with friends (Shantz, 1987). We also know that conflict between friends is more common than between age-matched classmates indicating that conflict resolution may be an important element of friendship development (Lollis et al., 1992). Over-management of children’s play during play dates may interfere with the development of effective conflict resolution skills leaving children less able to cope with normative conflict as they transition to school and limiting exposure to what is an important adaptive process. The experience of what has been described as constructive boredom (Louv, 2005) may also be precluded by high levels of parental supervision with children having fewer opportunities to creatively manage and monitor their own play experiences.

An important barrier preventing parents from fully adopting an open attention approach to supervision as described in the Vigilant Care model (Omer et al., 2016) or allowing meaningful transfer of responsibility from parent to child was the perceived expectations of the guest child and their family. For many parents play dates are times of effortful social presentation with a clear set of prescribed expectations and obligations. The perceived pressure to provide a fun and interesting experience for guests prevented parents from allowing fully autonomous play or allowing children to manage more difficult aspects of peer play independently. Although most parents endorsed the values of free play, and described children as preferring indirectly supervised play environments, parents reported ultimately feeling responsible for “balancing the whole thing” to ensure the play date was a success. This highlights an important conflict in the parental role during children’s play dates: to support autonomy and independence as well as ensure play is harmonious and enjoyable. This conflict contributed to parents’ feelings of ambivalence about play dates.

We know that children of parents who facilitate autonomous play are more likely to have positive peer relationships and are less likely to experience bullying (Healy et al., 2015) and that parents who promote the transfer of responsibility via sensitive scaffolding support the development of emotion regulation, task persistence and behavioral control (Neitzel & Stright, 2003). It is likely that prolonged parental assistance in peer relationships may inhibit the development of emotion regulation by restricting a child’s progression from passive to active agent and by perpetuating a view of the child as a helpless dependent. It will be important for future researchers to consider the barriers preventing parents from adopting open attention styles of supervision or sensitively transferring responsibility during play dates more consistently. Interventions to increase parental tolerance of minor conflict and boredom may be an important way to promote healthy social and emotional development at this age and should consider structural and social barriers, for example via perceived social pressures and expectations, as well as individual level attitudes of parents. Work with parents to facilitate free play during play dates should also acknowledge the social and cultural expectations for parents to play an active role in facilitating the enhancement of children’s social networks (Mose, 2016).

Parents identified common features of play dates suggesting that some aspects of play in this context may be similar, perhaps universal, for children of this age. The term ‘play date’ was described as having an operationalizing effect, establishing a clear set of norms and expectations, leading one parent to describe play dates as “carbon copies” of one another. Although parents reported frequent imaginative play during play dates, most play occurred inside in a relatively controlled play space with parents generally aware of what children were doing. The coinage of the term ‘play date’ is relatively new and coincides with a marked decrease in children’s autonomous play outdoors (Mose, 2016). Previous research indicates that there is a connection between the increase in formal play dates at home and fears for children’s safety (Mose, 2016). One consequence of this shift indoors is that children’s play environments are generally less diverse and more regulated than was the case for previous generations, with a risk that play dates may contribute to the sanitization of children’s play (Mose, 2016).

Although parents reported that time constraints, often due to structured extra-curricular activities, limited the number of play dates available to children, most parents reported making time to ensure children were able to experience at least some play dates, even when this came at a cost to the parent themselves. Parents were primarily motivated to arrange play dates because of the perceived social and emotional benefits for children. These benefits included social networking as well as child social skill development. We know that play with friends is essential for children’s development, particularly when unstructured and child led (Gleave & Cole-Hamilton, 2012). There has been a shift in recent years from predominantly unstructured play to predominantly structured social activities which has been associated with diminished opportunities for children to develop important social skills (Gray, 2011). It is likely that, as children’s access to unstructured play outdoors is diminished, play dates have become important opportunities for children to play semi-independently and to experience some sense of agency and autonomy necessary for social skill development. It will be important for future research to explore tensions between the perceived imperative for play dates to be safe, harmonious, managed, *and* child-led to determine the how parents might be best supported to promote positive play in this context.

Although all parents interviewed ascribed significant benefits to having play dates, access was not universal for all children and families. Parents identified a clear play date community with play dates common between like-minded families who share similar backgrounds and values, and less likely between families who do not have a pre-existing connection. These findings align with previous research demonstrating that play dates are strongly associated with social class divisions and that part of parents’ motivation to establish a play date community is the retention of social capital via intentional networking (Mose, 2016). Parents reported that children were often selected for play dates if they were perceived to be well-behaved or if parents felt they could be important social contacts. Although few respondents expressed an explicit motivation to exclude children who were not part of the play date community, children outside this network were thought to be less likely to receive play date invitations. Given the many benefits parents associated with play dates, including the development of strong classroom friendships and the opportunity to practise important social skills, exclusion from the play date community could make classroom integration more of a challenge for these children. One implication of this finding is that children perceived to have less developed social skills at school entry, or those perceived as having low social capital, may have restricted access to play contexts that support social development and so compound difficulties over time. Improved strategies to widen access to play dates, particularly for children vulnerable to exclusion, could be an important way to support and enhance the peer status of these children. However, democratizing access to play dates may be resisted by parents particularly after the establishment of secure friendship groups and social reputations within children’s networks. Consequently, interventions to promote access to play dates may be particularly important during the transition to school or during the first term when parents are “exploring different options” and access to play dates may be predicated in part on children’s perceived ability to “play nicely”. Early years’ strategies to support pre-school children to practise and navigate free play through play dates may be one way in which to enhance the social capital of vulnerable children prior to starting school and could include a more focused emphasis on schools and pre-schools supporting and encouraging play dates before and during the transition to school to give more children the opportunity to practise social play in domestic environments. This is likely to particularly important for current cohorts of children starting school whose social and emotional development, and play skills have been negatively impacted by Covid-19 related school closures and lockdowns (Tracey et al., 2022). Access to play dates may also be restricted by parental ambivalence and the extent to which parents’ value, prioritize, or are motivated to initiate play dates. Play dates are associated with additional stress and effortful social presentation which may limit access for some families, particularly parents with low confidence, or those anxious about how their home may be perceived.

Parents’ descriptions of children’s play during play dates suggest that they may be increasingly important contexts for children to engage in unstructured free play, and imaginative role play was cited as a particularly valued feature by many parents. The decline of imaginative free play outside of the home over the last 20 years, including during play outside and during the school day, has corresponded with increasing rates of childhood anxiety and depression (Sadler et al., 2018). This has led some researchers to hypothesize a link between a free play deficit and increased rates of childhood psychopathology (Gray, 2011). Researchers have called for increased opportunities for children to engage in self-directed play to support healthy social and emotional development (Tremblay et al., 2015). Results from this study indicate that play dates may be an important context for children to experience this kind of play, although this is likely to be limited by anxieties about children’s safety and a perceived pressure to manage and facilitate play.

Finally, parents identified that the quality of children’s play and typical levels of supervision change over time. Play dates during primary school were described as a window of opportunity for children to experience regular imaginative free play with their friends. During pre-school parents felt that children were too young to sustain prolonged periods of imaginative play whereas after the age of 8 or 9 screens and technology were cited as reasons for the decline of this kind of play. It may be that between the ages of 5-8 years play dates offer an important opportunity for children to play freely with their peers and to transport themselves imaginatively beyond the confines of the bedroom. This type of imaginative role-play has been associated with a range of benefits including Theory of Mind development via exposure to alternative representations and viewpoints (Schwebel et al., 1999) and development of emotion regulation (Galyer & Evans, 2001). Importantly parents of only children indicated that play dates were often their children’s only opportunity to engage in this sort of play.

Results from this study suggest that play dates are a rich and underexplored play context for young children, associated with significant cultural and social expectations. This study provides important qualitative insight for further play date research, in particular highlighting significant tensions about the role and purpose of play dates as perceived by parents. Play dates are not neutral play environments but are associated with significant, and often conflicting, expectations and priorities which may impact the quality and range of children’s play, as well as limit access to specific groups of children. Further research is needed to explore the extent to which play dates are environments for child-led free play, and the impact of parental management on children’s play. It will also be important for researchers to explore associations between play date frequency, quality of play and measures of children’s social and emotional competence to determine the likely impact of this play environment on children’s development.

There are a number of limitations to consider in relation to this work. Although data analysis identified common themes across the dataset, the relatively small sample size makes it likely that additional themes, complexities, and nuances may not have been captured in the current study. In particular, the current sample did not include any parents who had no, or very limited experience, of play dates. Given the strong associations with social networking, it will be important for future researchers to extend this qualitative work with parents who do not have play dates, or who are marginalized from the play date community; for example, parents of children who are not invited for play dates, or who themselves do not have social networks within the school. It will also be important for future research to explore the impact of the covid-19 pandemic on young children’s access to play dates, and any associated changes in parental management of play. Although the sample of parents and children in this study included a broad range of families in terms of siblings and socio-economic status, lack of ethnic diversity of parents is another important limitation. Future qualitative work with parents from a range of cultural and ethnic backgrounds will be important to establish whether play dates vary between demographic groups, or if there are any additional access barriers for families from different cultural or social backgrounds.

## Conclusion

This study provides qualitative evidence to support the inclusion of play dates into play research and offers an insight into a rich environment that could enhance understanding of children’s early social development. Results from this exploratory study suggest that play dates are an important and unique environment for children to experience social play and are associated with social and emotional skill development. Parents overwhelmingly value these experiences but also associate them with stress and anxiety. Play dates can also be exclusive with invitations made and received on the basis of perceived social networking value. This ambivalence and social networking motivation can restrict access for some families. Certain groups of children and families may find access to play dates challenging and could benefit from early years’ interventions to support children’s play skills to support integration into the network. Play dates offer children time for creative, imaginative play but parental monitoring and supervision may limit the extent to which children experience free play, and this may moderate the value of the experience.

## Supplementary Information

Appendix_JCFS
