# Supplementary material for: “Are They Going to Play Nicely?” Parents’ Evaluations of Young Children’s Play Dates
Source: J Child Fam Stud. 2022 Dec 13:1–14. Online ahead of print. doi: 10.1007/s10826-022-02499-4 (PMC9747075; doi:10.1007/s10826-022-02499-4)
Supplement: Supplementary file 1 — Appendix_JCFS [file 10826_2022_2499_MOESM1_ESM.docx]

**Appendix: Semi-structured interview schedule**

**Section 1: Defining terms**

1. What words do you and your family/friends use when arranging for your child to meet up with a friend outside of school?

- Briefly, what comes to mind when you think about [term introduced by parent]
- How often have you heard the term used?
- What parent/child factors do you associate with [term]

**Section 2: Evaluations**

1. How do you feel about play dates [or alternative term]?

- What is it about play dates that you enjoy or find challenging?
- Do you feel any differently about play dates at your home versus at another families’ home?
- Have you experienced any barriers that have made arranging them difficult? (e.g. social factors, time, location, child factors)
- Do you feel there is pressure or expectation to have play dates?

1. How important do you consider play dates to be for your child?

- How does your child feel about having play dates?
- How central are they to your child’s social experience?
- What skills in particular might they help your child develop?
- In what ways do you feel they have influenced your child’s friendships?
- Is it important for children to experience play dates before starting school?

1. How important do you think play dates are for parents?

- Have they changed your relationship with your child in any way?
- Have they changed your relationship with other people’s children?
- Have they changed your relationship with other parents?

**Section 3: Describing play dates**

1. Can you describe a typical play date that you have hosted?

- Who initiated/arranged?
- How did you decide who to invite?
- How frequently do they happen?
- How long do they last?
- Who attended?
- Where do they take place?
- What types of play/activities do the children engage in? Structured or unstructured?
- Do you structure the activities in any way?
- How closely do you supervise the children?

1. What do you usually do before and during a play dates?

- Do you prepare or structure activities in any way?
- How much does your child want you involved?
- What do you do if you child argues/falls out with the other child/ren?
- What does the other parent do?

1. How have play dates that your child has experienced away from home

differed from the kind of play dates you provide at home?

- Prompts as above

1. Thinking back to when your child first started having play dates, has anything changed compared to the play dates they have now?

- Frequency
- Range of friends
- Activities
- Supervision
- Approximately when did your child start having play dates?

1. Is there anything else you’d like to add about playdates in general, or about your child’s experiences in particular?
